# A randomized, controlled proof-of-concept trial evaluating durable effects of memory flexibility training (MemFlex) on autobiographical memory distortions and on relapse of recurrent major depressive disorder over 12 months

**DOI:** 10.1016/j.brat.2021.103835

**Published:** 2021-05

**Authors:** Caitlin Hitchcock, Alicia J. Smith, Rachel Elliott, Cliodhna O'Leary, Siobhan Gormley, Jenna Parker, Shivam D. Patel, Carlos V. Esteves, Evangeline Rodrigues, Emily Hammond, Peter Watson, Aliza Werner-Seidler, Tim Dalgleish

**Affiliations:** a Medical Research Council Cognition and Brain Sciences Unit, University of Cambridge, UK; b The Black Dog Institute, UNSW, Sydney, Australia; c University of Exeter, UK; d Cambridgeshire and Peterborough NHS Foundation Trust, UK

**Keywords:** Autobiographical memory, Depression, Relapse prevention, Randomised controlled trial, Cognitive therapy

## Abstract

Low-intensity psychological interventions that target cognitive risk factors for depressive relapse may improve access to relapse prevention programs and thereby reduce subsequent risk. This study provides the first evaluation of an autobiographical memory-based intervention for relapse prevention, to establish whether memory-training programs that are efficacious for acute depression may also aid those currently in remission. We also provide the longest follow-up to-date of the effects of autobiographical memory training on autobiographical memory processes themselves. This pre-registered randomized-controlled proof-of-concept trial (*N* = 74) compared an autobiographical Memory Flexibility (MemFlex) intervention to Psychoeducation about cognitive-behavioral mechanisms which maintain depression. Both interventions were primarily self-guided, and delivered via paper workbooks completed over four weeks. The key cognitive outcome was ability to retrieve and alternate between specific and general autobiographical memories. Co-primary clinical outcomes were time until depressive relapse and depression-free days in the twelve-months following intervention. Results indicated a small-moderate effect size (*d* = 0.35) in favor of MemFlex for the cognitive outcome. A small Hazard Ratio (1.08) was observed for time until depressive relapse, along with a negligible effect size for depression-free days (*d* = 0.11). Although MemFlex produced long-term improvement in memory retrieval skills, there was little support for MemFlex as a relapse prevention program for depression.

## Editor highlights

• Study evaluates autobiographical memory-based intervention for relapse prevention.
• Intervention-driven gains in autobiographical memory were maintained one year later.
• MemFlex did not reduce risk of depressive relapse any more than Psychoeducation.
• Autobiographical memory interventions may be best utilised as treatment programs.

Recurrent depression is a leading worldwide cause of disability (World Health Organisation, 2017), imposing a significant financial burden on global economies, and a large personal cost for individuals, communities, and society. Reducing the recurrence of depression is therefore a key priority. Cognitive risk factors are one of the strongest predictors of depressive relapse, and are one of the few risk factors amenable to psychological intervention. Development of low-cost, low-intensity intervention programs that reduce cognitive risk and can be delivered upon discharge from current treatment services may thereby reduce subsequent relapse and costs to both individuals and healthcare systems.

Translation of basic cognitive science into clinical practice offers a rich avenue for the development of such programs. There is considerable research demonstrating that depressive prognosis is predicted by the ability to access autobiographical memories about the personal past. Autobiographical memory provides the foundation for our sense of self (Conway & Pleydell-Pearce, 2000; Hitchcock & Dalgleish, submitted) and shapes the beliefs we hold about ourselves (Hitchcock, Rees, & Dalgleish, 2017). Disruptions to retrieval of memories from this system have been demonstrated to consistently predict depressive relapse, up to a year later, over and above the effect of current symptom levels (recent meta-analysis by Hallford, Rusanov, Yeow, & Barry, 2020; updating that of Sumner, Griffith, & Mineka, 2010). Critically, autobiographical memory distortions do not improve when depressive symptoms remit (e.g., Semkovska et al., 2019). While initial research in this area (see Williams et al., 2007) posited that it was the ability to retrieve specific memories of single incident events which drives emotional disturbance, more recent evidence has suggested that autobiographical memory distortions in depression may be better characterized as an impaired ability to flexibly move between specific and general levels of memory representation (Dalgleish et al., 2007; Dritschel, Beltsos, & McClintock, 2014; Hitchcock et al., 2019).

Both specific memories (e.g., *dinner with my partner last night*) and generalized memories which link categories of events (e.g., *spending time with my partner*) are used in the service of other cognitive operations which underlie daily functioning, particularly in the context of recovery from depression. Specific memories aid regulation of transient low mood (Joormann & Siemer, 2004) and provide a blueprint for imagining and planning for the future (Jing, Madore, & Schacter, 2017), while general memories encode regularities of experience to inform judgments about ourselves and the world around us (Klein, Cosmides, Tooby, & Chance, 2001). Intervention to improve the ability to flexibly move between different memory types has therefore been demonstrated to improve depressive symptoms (Hitchcock, Gormley, Rees, et al., 2018; Hitchcock et al., 2016), with some evidence to suggest that improving memory flexibility may also aid treatment of psychosis (Edwards, Garety, & Hardy, 2020) and posttraumatic stress disorder (Moradi et al., 2020). Here, we were interested in whether improving memory flexibility in those remitted from depression may decrease risk of future relapse.

The Memory Flexibility (MemFlex) intervention was developed from basic science to provide a low-intensity, low-cost intervention that is mechanism-focused, rather than symptom-focused. Building upon the success of prior autobiographical memory programs (e.g., Memory Specificity Training; for meta-analysis see Barry, Sze, & Raes, 2019), MemFlex aims to improve retrieval of, and flexible movement between, both specific and general memory types. In addition, the intervention seeks to ameliorate the negative memory bias associated with depression. When formerly depressed individuals do recall positive information, it is less vivid and evokes a weaker emotional response (Werner-Seidler & Moulds, 2011), thereby reducing the ability to use positive memories to regulate low mood (Speer & Delgado, 2017) – a skill which is critical to reducing relapse within diathesis-stress models (A. T. Beck, 1967; Robins & Block, 1989). Reduced access to positive autobiographical information and repeated recall of generalized, negative memories about the past may also reinforce the negative self-schemata that drive chronic depressive presentations (Beck, 1967; Hitchcock & Dalgleish, submitted). Indeed, improving vividness and accessibility of positive autobiographical episodes yields improvement in depressive symptoms (for meta-analysis see Hitchcock, Werner-Seidler, Blackwell, & Dalgleish, 2017). Simultaneously targeting negative memory bias and the ability to flexibly move between memories may therefore have the added benefit of reducing compounded effects of these two depressogenic risk factors.

Exploratory trials of MemFlex with those who are acutely depressed have demonstrated significant improvement in both memory flexibility and depression (Hitchcock, Gormley, Rees, et al., 2018; Hitchcock et al., 2016). Our recent randomized controlled trial with currently depressed participants (Hitchcock, Gormley, Rees, et al., 2018) compared MemFlex to Psychoeducation, and demonstrated a large between-group effect size in favor of MemFlex for improvement in memory flexibility (*d* = 1.03). In addition, those completing MemFlex experienced, on average, an additional two weeks free from depression over the three months following intervention completion, relative to those who completed Psychoeducation. Based on these findings, promising results in our pilot evaluation of MemFlex in a remitted sample (Hitchcock et al., 2016), and the significant literature indicating that autobiographical memory processes predict depressive relapse, this study sought to determine whether MemFlex may have potential as a relapse-prevention program for depression. The workbook-based, self-guided format of MemFlex is likely to be deliverable for a lower-cost and shorter time commitment than current gold-standard relapse prevention programs (e.g., mindfulness-based cognitive therapy (MBCT); National Institute for Health and Care Excellence, 2009). The delivery format of MemFlex involves a single 30–45 min orientation session with a facilitator who does not require any specialist training, followed by an entirely self-guided workbook completed over four weeks, dramatically reducing the resourcing and training needed for delivery relative to other relapse prevention interventions.

This proof-of-concept trial therefore estimated the effect size of MemFlex on the recurrence of depression over a twelve-month period, relative to an active control condition – Psychoeducation. In assessing autobiographical memory a year after intervention had ceased, this trial also sought to provide the longest-to-date evaluation of the durability of any intervention-driven changes in autobiographical memory distortions. Determining that autobiographical memory-based intervention can produce durable, long-term change in this trait-like risk factor is critical to evaluating the efficacy of such interventions. In line with recommendations for the phase-based development and evaluation of novel interventions (Craig et al., 2008), this early-stage, randomized controlled trial had the primary aim of estimating the likely effect size of MemFlex on measures of depressive relapse, to inform a later-stage, fully-powered trial. Interpretations are therefore drawn from effect sizes, not significance tests, which would be the focus of any subsequent later-stage trial. We hypothesised effect sizes in favor of MemFlex, such that individuals in remission from Major Depressive Disorder who completed MemFlex would experience a greater number of depression free days in the twelve months following workbook completion and demonstrate a longer period of time until depressive relapse, compared to those who completed Psychoeducation.

## 1 Method

### 1.1 Protocol registration and publication

CONSORT standards (Altman et al., 2001) were adhered to in study design and completion. The published trial protocol presents full study methods (Hitchcock, Gormley, O'Leary et al., 2018). This trial was pre-registered on clinicaltrials.gov (NCT02614326).

### 1.2 Study design

**Fig. 1:**
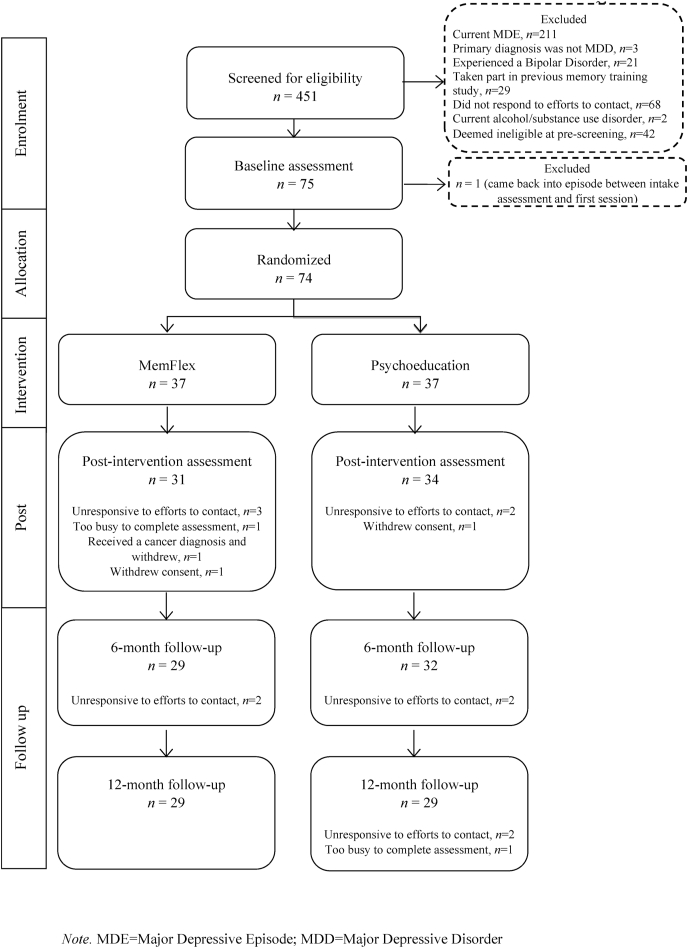
CONSORT diagram of study participation. Note. MDE = Major Depressive Episode; MDD = Major Depressive Disorder.

We conducted a single-blind, participant-level randomized controlled trial comparing MemFlex to Psychoeducation. Participants completed a four-week program comprising an initial face-to-face session introducing either the MemFlex or Psychoeducation materials, followed by eight self-guided workbook sessions. Assessments were completed at baseline, post-intervention, six month follow-up, and at twelve month follow-up (the primary clinical endpoint). The CONSORT diagram is presented in Fig. 1.

### 1.3 Participants and recruitment

In accordance with recommendations for the evaluation of complex interventions (Medical Research Council, 2000), this early-stage randomized controlled trial aimed to generate estimates of the range of likely effect sizes of MemFlex (relative to Psychoeducation) on our primary outcomes. Twenty five participants per condition will provide a plausible range of point estimates. Given the long-term follow-up we implement, we aimed to recruit 35 participants in each condition, and we obtained a final sample size of 74.

Participants were recruited in Cambridge, UK, from the MRC Cognition and Brain Sciences Unit research volunteer database, local mental health services, and through advertisements in the general community (e.g., posters in General Practice surgeries). Inclusion criteria were a minimum age of 18 years and a primary diagnosis of Major Depressive Disorder (MDD), with all participants in partial or full remission as determined by the fifth edition of the Structural Clinical Interview for the DSM disorders (SCID; First, Williams, Karg, & Spitzer, 2015). All SCIDs were second-rated by a clinical psychologist, who agreed on diagnosis for 100% of cases. Exclusion criteria were experience of another mood disorder (e.g., Bipolar Disorder), psychosis, personality disorders, current drug or alcohol use disorder (determined by the SCID), or significant cognitive impairment (assessed through self-report). Participants were free to continue any other medication or psychological interventions whilst participating in the study.

### 1.4 Interventions

**MemFlex.** The MemFlex program is a self-guided workbook which aims to improve key memory skills including the ability to flexibly move between specific and general personal memories, and attenuate negative memory bias. The workbook achieves this through utilising cued-recall tasks to both positive and emotionally neutral cues, introduced to participants in an initial 30–45 min face-to-face session with a researcher. In the face-to-face session, the researcher outlined the different types of autobiographical memories (e.g., specific memories and generalized themes) and their role in role in everyday functioning and depression. The researcher assisted each participant with practice exercises, and provided feedback to ensure that they fully understood the tasks. The participant then completed eight workbook sessions over the following four weeks. Participants received a phone call from the researcher half way through the four week self-guided period to address any difficulties or questions they may have had with the workbook. Participants were encouraged to contact the researcher if they experienced any problems, and approximately 10% of participants did so to confirm that the memories they were recalling were appropriate.

The first MemFlex workbook sessions focused on reducing bias away from the retrieval of negative and general memories, towards improved access to specific memories of positive and neutral valence. Participants were required to provide memories in response to positive or neutral cue words. Subsequent sessions aimed to improve the reduced vividness of positive memories experienced in depression by encouraging participants to elaborate on specific details. This again involved using repeated cued-recall exercises. The final workbook sessions sought to promote flexible movement between memory types. Participants were required to identify individual event memories that group together to form a general theme, as well as explicitly identify specific event memories that contribute to a nominated theme.

Fifteen percent of initial session audio recordings were reviewed to ensure fidelity to the intervention manual. Adherence was 100% for 61% of recordings, and 89% for 39% of recordings. For those with 89% adherence, facilitators did not assist participants to set a schedule for workbook completion, and in one case, some practice exercises were not completed.

**Psychoeducation.** Participants completed an eight session workbook over a four week period (i.e., programs were matched for time commitment). As in the MemFlex condition, participants attended an initial 30–45 min face-to-face session in which the researcher introduced the workbook and provided psychoeducation on the causes of depressive relapse. Fifteen percent of initial session audio recordings were reviewed to ensure fidelity to the intervention manual. Adherence was 100% for 66% of recordings, and 89% for 33% of recordings. For those with 89% adherence, facilitators did not assist participants to set a schedule for workbook completion. Each participant received a call from the researcher halfway through the four-week self-guided period to check progress and clarify any problems they may have been having with the workbook material. Again, participants were encouraged to contact the researcher if they experienced any difficulties with the workbook, and approximately 10% of participants did so to clarify workbook content.

The Psychoeducation program content closely replicated that of the psychoeducational material provided by UK NHS psychology services. The workbook, adapted from that previously used in a trial for those with MDD (Hitchcock, Gormley, Rees, et al., 2018), aimed to provide a rigorous comparison condition that is representative of current low-intensity intervention options. Each session provided information on different cognitive and lifestyle mechanisms that perpetuate depression and persist between episodes. These mechanism are commonly addressed in cognitive behavioral therapy (CBT) and are targeted in preventative cognitive therapy which has been shown to reduce the occurrence of depressive relapse for up to ten years (Bockting et al., 2015). Addressed mechanisms included worry, perfectionism, poor sleep patterns, interpersonal difficulties, and anger. The first workbook session reviewed material covered in the initial face-to-face session. Subsequent sessions 2–7 provided further information on what maintains each mechanism, techniques for improving the mechanism, and exercises which encouraged participants to reflect on the role of that mechanism in their own life and depressive experience. At the end of each session, four multiple-choice questions were delivered to ensure engagement with the material and match session engagement to the MemFlex session. Session 8 required participants to reflect on what they had learnt and make a plan to integrate their learning with their own personal strategies to create a relapse prevention plan.

### 1.5 Measures

**Primary clinical outcomes.** The co-primary clinical outcomes were number of depression free days from post-intervention to twelve-month follow-up, and time until depressive relapse until 12 month follow-up, both measured using the Longitudinal Interval Follow-up Evaluation (LIFE) for the SCID (Keller et al., 1987). All LIFE assessments were reviewed by the supervising clinical psychologist who was blind to intervention allocation. In the case of disagreement with the original assessor, the clinical psychologist re-administered the LIFE. One hundred percent agreement was obtained between raters for the final data.

**Secondary clinical outcomes.** The secondary clinical outcomes were depressive symptoms at twelve month follow-up, as indexed on the Beck Depression Inventory II (BDI-II; Beck, Steer, & Brown, 1996), and depressive status (i.e., presence or absence of a major depressive episode) at twelve month follow-up, again measured using the LIFE.

**Cognitive outcome.** Our key cognitive outcome was maintained change in memory flexibility from baseline to post treatment and across the twelve month follow-up period, indexed on the Alternating Instructions version of the Autobiographical Memory Test (AMT-AI; Dritschel et al., 2014) as change in the total number of correct memories recalled. The AMT-AI is a cued recall task which requires individuals to recall either specific event memories or general memories (which summarise a category of events) in response to positive, negative and neutral cue words. Participants were instructed to recall specific memories to a block of six cue words (specific block), general memories to a block of six cue words (categoric block), and retrieve specific and categoric memories in alternation to a block of twelve cue words (alternating block). The order in which these blocks were presented was randomized, and cue words were randomized to block. Four word lists were counter-balanced between assessments, stratified by intervention allocation, for each participant. An instruction to recall a specific or general memory was presented on the computer screen followed by a cue word and participants were given 1 min to retrieve and verbally report the memory. The researcher coded responses as specific (i.e., an event that happened on a single occasion), general/categoric (i.e., an event that happened on multiple occasions), extended (i.e., an event lasting longer than a day), repeated (i.e., a memory that has already been reported), semantic associate (i.e., information related to the cue that is not a memory), or an omission (i.e., the participant could not recall a memory). Inter-rater reliability was strong for the number of correct memories, intraclass correlation coefficient = 0.85. As in studies validating the AMT-AI (e.g., Hitchcock et al., 2019), we used the proportion of correct responses (with omissions excluded) as our primary outcome.

**Treatment expectancy and acceptability.** At the initial session, the Credibility Expectancy Questionnaire – Patient Version (Devilly & Borkovec, 2000) was administered to both conditions to determine treatment acceptability. At post-intervention, the researcher also checked the number of workbook exercises that had been completed to assess participant engagement with intervention materials and adherence to the program.

**Additional process measures.** We also assessed a number of cognitive-behavioral process factors that may mediate the effect of MemFlex on our primary and secondary clinical outcomes, to inform selection of mechanism measures for any later trial. Social problem solving was indexed on the short version of the Means End Problem Solving Task (Platt & Spivack, 1975). The task measured individuals' ability to identify strategies for overcoming a given interpersonal problem (e.g., not getting along with your boss at work). Participants were presented with a scenario and verbally reported their answers which were audio recorded for later transcription and scoring. Outcomes were the number of means (i.e., steps) given to achieve the goal and the likely effectiveness of the given approach, scored by a blinded rater. Inter-rater reliability was acceptable for both means, intraclass correlation coefficient = 0.83, and efficiency, intraclass correlation coefficient = 0.78. The Verbal Fluency Task (VFT; Tombaugh, Kozak, & Rees, 1999) assessed the fluent retrieval of verbal information. Finally, rumination was assessed using the Rumination Response Scale (Lyubomirsky & Nolen-Hoeksema, 1995) and cognitive avoidance was measured using the Cognitive Avoidance Questionnaire (Sexton & Dugas, 2008). The Ruminative Response Scale consists of 22 items that measure the tendency to ruminate in relation to low mood. Internal consistency was excellent in the current study, α = 0.95. The Cognitive Avoidance Questionnaire consists of 25 items that index thought substitution and suppression, distraction, avoidance of threatening stimuli, and transformation of images into thoughts. Internal consistency was excellent in the current study, α = 0.90. The Digit Span working memory test (Wechsler, 2010) was also administered at baseline to control for any baseline differences in memory function between the groups.

### 1.6 Procedure

Ethics approval was obtained from the NHS National Research Ethics Committee (East of England, 11/H0305/1). All individuals provided informed consent prior to participation. Participants were randomized to condition by the trial statistician (Watson) using a computer-generated random number allocation, and initial session facilitators were informed of treatment allocation after completion of the baseline assessment by opening a sealed opaque envelope. The workbook introduction was subsequently completed. Cognitive target, clinical outcomes and process measures were assessed at baseline, post and six month (6 m) follow-up assessments. Only the AMT-AI and LIFE were administered at the final twelve month (12 m) assessment. For 12 m follow-ups occurring during the COVID-19 pandemic, restrictions to face-to-face testing saw that only the LIFE was administered over the telephone, as the AMT-AI cannot be delivered over the telephone. Assessment materials were counterbalanced between each assessment and all assessors were blind to treatment allocation. Participants were reimbursed GBP£6 per hour for their time, as well as subsidy for their travel. All risk issues were managed by the trial co-ordinator (Hitchcock), a Clinical Psychologist. No adverse events were reported.

### 1.7 Statistical analysis approach

All analyses were pre-specified in the published trial protocol (Hitchcock et al., 2019). Analyses were completed by the trial statistician (Watson) who was blind to study hypotheses. We report intent-to-treat analyses (and thus intent-to-treat means) for our primary and secondary outcomes using multiple imputation for missing data, applying Satterthwaite's correction. The number of imputations reflected the proportion of missing data for that variable (Bodner, 2008). ANOVAs yielded an *F* or *t* value which was used to evaluate between-group differences on the cognitive target, treatment credibility and process outcomes. Cohen's *d* and associated 95% confidence intervals were calculated from the *t*/*F* value. Hazard ratios are reported from a Cox survival analysis evaluating our co-primary clinical relapse outcome. As this was an early-stage proof-of-concept trial, conclusions regarding potential treatment effects should be drawn from the Hazard ratios and Cohen's *d* (and associated confidence intervals), rather than presence or absence of statistical significance. Inferential statistics are reported for the reader's interest only.

## 2 Results

### 2.1 Sample characteristics

Baseline means for participant demographics, and cognitive and clinical outcomes are presented in Table 1. Approximately 28% of the participants had experienced ‘too many depressive episodes to count’, as indexed on the SCID. Comorbid diagnoses were experienced by 54.1% of participants, and included Generalized Anxiety Disorder (27.0%), Posttraumatic Stress Disorder (5.4%), Panic Disorder (4.0%), Social Anxiety Disorder (4.0%), Obsessive Compulsive Disorder (2.7%), and eating disorders (2.7%). There was no significant difference between conditions in either the number of comorbid diagnoses, t(60) = 1.33, p = .19, or prior depressive episodes, t(59) = 0.26, p = .79. No significant between-group differences were observed for demographic or concurrent treatment variables (see Table 1). At baseline, the mean proportion of correct responses on the AMT-AI was 0.68 (SD = 0.18), 95%CI [0.64, 0.72], which was slightly higher than we have previously observed in a currently depressed samples (0.62, 95% CI [0.61, 0.63]; Hitchcock et al., 2019), but as expected lower than that observed in a never-depressed sample (0.83, 95% CI [0.77, 0.89]; Hitchcock et al., 2019).

**Table 1:** *Mean (SD) participant characteristics at baseline*.

	MemFlex (*n* = 37)	Psychoeducation (*n* = 37)
Age	42.71 (14.70)	45.49 (15.58)
Number of females	23	24
Caucasian (%)	83.78	91.89
Education history	1; 6;16; 9;5	3; 5;16; 7;6
Currently employed (%)	51.35	56.76
Number of prior episodes	9.40 (7.91)	9.93 (7.86)
Number of comorbid diagnoses	0.87 (1.17)	0.53 (0.80)
Current Psychological treatment (%)	29.7	21.6
Current medication (%)	73.0	51.4
Digit Span	18.49 (4.35)	17.68 (3.93)
Beck Depression Inventory-II	16.11 (10.48)	16.22 (10.31)
Proportion correct on AMT-AI	0.67 (0.17)	0.69 (0.19)

Note. *p < .05. AMT-AI = Autobiographical Memory Test-Alternating Instructions. For education history, highest level of UK education is 5th form; 6th form; undergraduate degree; postgraduate degree; diploma or professional training.

### 2.2 Intervention acceptability and feasibility

There was equivalent attrition between conditions, with eight participants per arm lost by 12 m follow-up (see Fig. 1). We achieved good adherence for a self-guided intervention, with 83.8% of MemFlex participants and 91.9% of Psychoeducation participants completing all eight workbook sessions. Non-completers were those who did not attend the post assessment, and as such, we were unable to record how many workbook sessions they had completed.

Both interventions were perceived to be acceptable according to participant ratings on the Treatment Expectancy and Credibility Questionnaire. A multivariate ANOVA indicated no significant between-group difference and a negligible effect size for how logical the intervention seemed (all out of 9; MemFlex M = 7.81, SE = 0.20; Psychoeducation M = 7.74, SE = 0.20), how successful participants expected the intervention to be (MemFlex M = 6.03, SE = 0.24; Psychoeducation M = 6.40, SE = 0.24), how confident they would be in recommending the intervention to a friend (MemFlex M = 6.50, SE = 0.31; Psychoeducation M = 6.83, SE = 0.31, and the percentage (out of 100) improvement they expected in their symptoms (MemFlex M = 50.0, SE = 3.67; Psychoeducation M = 51.14, SE = 3.73, *F*(1, 69) = 0.10, *p* = .76, *d* = 0.07 [-0.40, 0.54].

### 2.3 Cognitive target

**Fig. 2:**
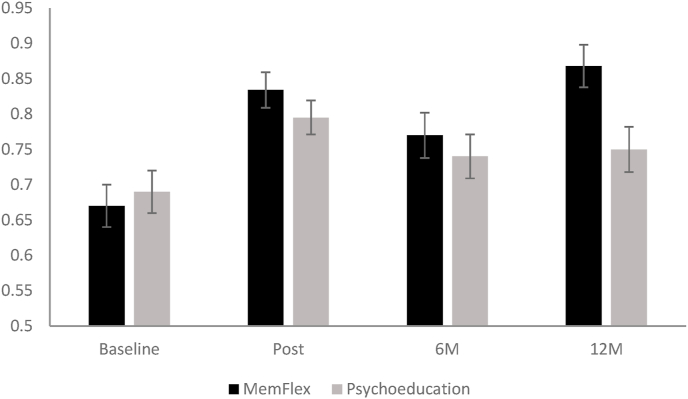
Mean proportion correct on the Autobiographical Memory Test-Alternating Instructions by intervention condition and assessment point (baseline, post-intervention, six month follow-up, and twelve month follow-up). Error bars are standard error of the mean.

We first assessed the intervention × time (baseline, post, 6 m follow-up and 12 m follow-up) interaction when predicting the proportion of correct memories on the AMT-AI (see Fig. 2; means in Supplementary Material), evaluating both linear and quadratic effects. A significant, linear interaction indicated a moderate effect size in favor of MemFlex, *F*(1, 174.39) = 4.91, *p* = .028, *d* = 0.51 [0.04, 0.99]. The effect size for the quadratic interaction term was negligible and non significant, *F*(1, 163.41) = 0.13, *p* = .72, *d* = 0.08 [-0.39, 0.55]. The effect size for time (i.e., improvement in AMT-AI performance from baseline to 12 m follow-up) was almost twice as large in the MemFlex condition, *F*(3, 55.14) = 12.27, *p* < .001, *d* = 0.81 [0.32, 1.30], than the Psychoeducation condition, *F*(3, 59.49) = 4.06, *p* = .01, *d* = 0.47 [0.01, 0.95].

At the primary cognitive end-point of post-intervention, the between-group effect size was trivial, *F*(1, 72) = 0.30, *p* = .59, *d* = 0.13 [-0.34, 0.60]. At 12 m follow-up (controlling for baseline scores), which was our primary clinical end-point, a moderate effect size in favor of MemFlex suggested a higher proportion of correct memories relative to Psychoeducation, *F*(1, 72) = 6.90, *p* = .011, *d* = 0.61 [0.13, 1.09], indicating that training effects were evident one year after intervention completion.

Intent-to-treat means for each block of the AMT-AI are presented in the Supplementary Materials. Exploration of the linear and quadratic effects of intervention on the different blocks of the AMT-AI indicated a small effect size for the linear intervention × time (baseline, post, 6 m follow-up and 12 m follow-up) interaction on proportion correct in the alternating block, *F*(1, 176.47) = 2.41, *p* = .12, *d* = 0.36 [-0.11, 0.84]. The effect size for improvement in performance from baseline to 12 m follow-up was almost twice as large in the MemFlex condition, *F*(3, 54.03) = 7.42, *p* < .001, *d* = 0.63 [0.15, 1.12], than the Psychoeducation condition, *F*(3, 58.03) = 2.33, *p* = .08, *d* = 0.35 [-0.12, 0.83]. Smaller effect sizes for the interaction were observed on the specific and categoric block, *F*s < 1.3 ds<0 .26.

### 2.4 Co-primary clinical outcomes

**Fig. 3:**
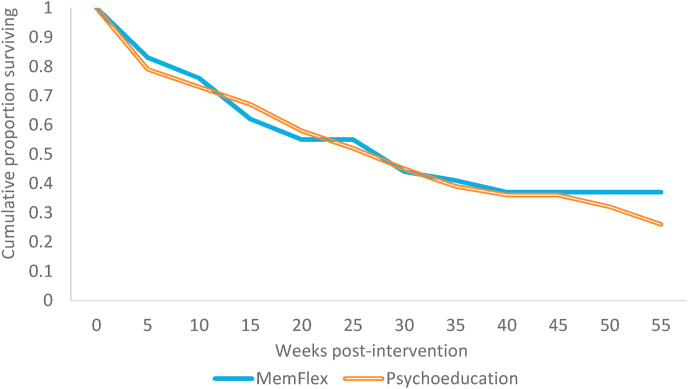
Survival curves for each intervention condition, where proportion surviving represents those with no depressive relapse across the 12-month follow-up period.

Our co-primary clinical outcomes were depression free days and time (from post-intervention) until relapse, up to the 12 m follow-up (see Table 2). Cox survival analysis indicated that, although the direction of the effect was in favor of MemFlex such that there was a higher risk of poor outcome in the Psychoeducation condition (Fig. 3), Hazard Ratio = 1.08 [0.59, 1.97], *p* = .81, the effect size was of a trivial magnitude, Cohen's *d* = 0.06 (Azuero, 2016).

**Table 2:** *Intent-to-treat relapse characteristics and clinical status at* 12-month *(12m) follow-up, by intervention condition*.

	MemFlex *(n* = 37)	Psychoeducation *(n* = 37)
Mean (SE) proportion of depression free days over 12 m	0.77 (.06)	0.81 (.06)
Mean (SE) duration in weeks of first relapse episode	4.07 (1.10)	3.64 (1.28)
Mean (SE) number of weeks until first relapse occurred	13.38 (2.79)	17.48 (3.28)
Number who relapsed over 12 m period	22.8	25.8
Number in episode at 12 m follow-up	6.7	6.6
Mean (SE) BDI-II at 12 m follow-up	12.84 (2.70)	15.28 (3.22)

*Note.* SE = standard error. Use of intent to treat data saw that means were averaged across multiple imputations, hence the non-whole numbers for number who relapse and number in episode.

There was a similarly negligible effect size for the between-group difference in the mean proportion of depression-free days from post-intervention until 12 m follow-up, *t* = 0.48, *d* = 0.11 [-0.36, 0.58], *p* = .63. Converting Hazard Ratios to Cohen's *d* allowed us to compare the treatment effect to that observed in our prior trial of MemFlex for patients experiencing an acute episode of depression (Hitchcock, Gormley, Rees, et al., 2018). The between-group effect size was smaller than that observed on depression free days in the prior trial with an acutely depressed sample (*d* = 0.36; Hitchcock, Gormley, Rees, et al., 2018).

### 2.5 Secondary clinical outcomes

Our secondary clinical outcomes were depressive status (currently in episode or still remitted) at 12 m follow-up and self-report symptoms on the BDI-II at 12 m follow-up (see Table 2). Again, a negligible effect size was observed between-groups in the number of participants experiencing a current major depressive episode at 12 m follow-up, *t* = 0.10, *d* = 0.02 [-0.45, 0.49], *p* = .92, along with a very small effect size for BDI-II scores, *t* = 0.71, *d* = 0.17 [-0.31, 0.64], *p* = .48. Overall, all participants experienced a moderate-large decrease in BDI-II scores from baseline to 12 m follow-up, *F*(3, 60.94) = 10.03, *p* < .001, *d* = 0.74 [0.25, 1.22].

### 2.6 Additional process measures

Finally, a series of intervention type × time interactions were completed on our additional process measures (means presented in Supplementary Materials). A negligible effect size for the interaction was observed for rumination, *F*(3,54.29) = 0.41, *p* = .74, *d* = 0.15 [-0.32, 0.62]. Small, non-significant effect sizes were observed in favor of MemFlex for cognitive avoidance, *F*(3,58.77) = 1.38, *p* = .26, *d* = 0.27 [-0.20, 0.75], and the number of retrieval errors made on the Verbal Fluency Task, *F*(2,65.33) = 1.54, *p* = .22, *d* = 0.29 [-0.33, 0.61]. Although a small effect size was found in favor of Psychoeducation for the number of steps produced in problem solving, *F*(2, 59.69) = 2.35, *p* = .10, *d* = 0.36 [-0.12, 0.83], a negligible effect size in favor of Psychoeducation was observed for the efficacy of that problem solving, *F*(2,63.02) = 0.25, *p* = .78, *d* = 0.12 [-0.36, 0.59].

### 2.7 Pre-registered exploration of mediation and moderation

Finally, we explored whether baseline memory performance or symptom characteristics influenced intervention effects on risk of relapse. Product terms were computed for intervention type × baseline performance on the AMT-AI, baseline BDI-II score, and number of prior depressive episodes, which were then (Cox) regressed on the hazard ratio. Neither the product term for baseline memory performance, *b* = -0.49, SE = 0.87, *p* = .58, nor the product term for baseline symptom severity, *b* = -1.38, SE = 0.93, *p* = .14, nor the product term for number of prior episodes, *b* = -0.15, SE = 0.84, *p* = .86, predicted relapse.

Finally, we explored whether the post-intervention score for total proportion of correct memories on the AMT-AI mediated the effect of intervention on the hazard ratio. No significant mediation was observed, Sobel test z = 0.63, *p* = .53.

## 3 Discussion

This randomized controlled platform trial aimed to estimate the likely size of the effect of MemFlex on risk of depressive relapse and on a cognitive-behavioral process with a known association with increased risk of relapse, relative to a current evidence-based low-intensity intervention – Psychoeducation. Moderate effect sizes in favor of MemFlex indicated that the intervention did improve our cognitive target of deliberate autobiographical memory retrieval, relative to Psychoeducation. That improvement was maintained one year later. This significantly extends the literature regarding the durability of memory changes produced by autobiographical memory-based interventions, as prior to this point, the longest follow-up evaluation had only been six-months post-intervention (Barry et al., 2019; Hitchcock, Werner-Seidler et al., 2017). Evidence of durable shifts in a trait-level depressive risk factor provides firm support for the efficacy of autobiographical memory-based interventions on the targeted cognitive mechanism of action. The larger between-group effect size at twelve month follow-up, relative to post-intervention, may indicate that time (and/or practice) may help to consolidate the new memory skills. Intervention-driven change in autobiographical memory did appear to be associated with improvement in some cognitive skills necessary for daily functioning and depressive recovery. We observed small, though as expected non-significant, effect sizes in favor of MemFlex for reductions in cognitive avoidance and improved fluency of retrieval of verbal information. However, results suggested that MemFlex would be unlikely to outperform current relapse prevention programs in reducing our primary clinical outcomes of depressive relapse rates or long-term symptomatology. Negligible effect sizes were found for the difference between Psychoeducation and MemFlex in the time to relapse and the number of depression-free days in the twelve months following intervention completion.

Relative to the treatment effects that MemFlex has demonstrated in samples with current MDD (Hitchcock, Gormley, Rees, et al., 2018) and posttraumatic stress disorder (Moradi et al., 2020), our results suggest that MemFlex may not be as efficacious for relapse prevention. It is worth noting that relative to Psychoeducation, MemFlex has previously produced small effects on remission rates (*d* = 0.24) in currently depressed individuals (Hitchcock, Gormley, Rees, et al., 2018), although a slightly larger effect size (*d* = 0.36) was observed for depression-free days in the follow-up period. Small effect sizes on depressive outcomes, relative to an active control condition of a currently used intervention, may therefore be expected. Small improvements above current interventions can still improve daily living for service-users (Funder & Ozer, 2019). Indeed, we did observe small follow-on effects for improvement in some daily cognitive skills. However, the negligible effect sizes observed in the current study suggest that further evaluation of MemFlex is unlikely to significantly improve treatment efficacy beyond current relapse prevention options.

A key strength of this study was use of a stringent comparison condition. The Psychoeducation intervention provided detailed information and techniques for managing cognitive-behavioral mechanisms that perpetuate depression (e.g., interpersonal difficulties, rumination and worry, perfectionism, sleep) and are commonly targeted in CBT and preventative cognitive therapy. The Psychoeducation condition also involved writing a relapse prevention plan (which may relate to the larger effect size for improvement in problem solving means in this condition). The Psychoeducation intervention was therefore more actively focused on what the participant could do to reduce their chances of relapse. The memory-based intervention, which does not directly address depressive symptoms, was as acceptable to participants, and as feasible in delivery, to a routinely-used low-intensity cognitive behavioral intervention. The availability of a range of evidence-based intervention options offers choice to patients, and completion of a memory-focused program which aims to enhance positive mental and emotional experiences may be of interest to some individuals who have not responded well to the more traditional CBT approach. Indeed, this was reported in the patient and public engagement activities we completed in conjunction with this trial. However, we did not include a measure of treatment preference in the current study, which is a limitation of the study. Shorter interventions that require less patient time and effort (i.e., relative to Mindfulness-Based Cognitive Therapy, our current gold-standard psychological relapse prevention program, which requires daily practice; National Institute for Health and Care Excellence, 2009) may also improve patient engagement and treatment completion rates. Further exploration of patient preference for relapse prevention programs may indicate whether further evaluation of MemFlex could improve the range of intervention options available to chronically depressed individuals who are currently remitted.

As this study was the first controlled trial to examine an autobiographical memory-based intervention for relapse prevention (rather than treatment), our findings could indicate that improving autobiographical memory distortions may only impact depressive symptoms when the depressogenic schemas (e.g.*, I am worthless*) that are supported by biased autobiographical memory retrieval (Hitchcock, Rees, & Dalgleish, 2017) are currently active. Models of autobiographical memory emphasize that autobiographical information underlies the self-concept (Conway & Pleydell-Pearce, 2000). If this is the case, training an individual to move away from retrieval of negative, generalized memories about the self may only be useful for improving emotional wellbeing when negative self-models are active and form the current, dominant model of self, as is the case in acute depression. Cognitive theories of recurrent depression (Beck, 1967) posit that depressogenic self-schema become dormant when depressive symptoms remit, and thus, improving the ability to access (positive and specific) information which contradicts depressogenic self-schema content may only be useful for reducing symptoms when that schemas are the current dominant model of the self. This would be consistent with purely autobiographical memory-focused intervention yielding treatment effects (Barry et al., 2019; Hitchcock, Werner-Seidler et al., 2017) but not relapse prevention effects for depression.

These findings also raise important questions regarding whether impaired autobiographical memory retrieval does predict relapse of depression in those who complete therapeutic interventions. Prior meta-analyses demonstrating significant, albeit small, effects of reduced memory specificity on future depressive symptoms (Hallford et al., 2020; Sumner et al., 2010) have not included treatment-completing samples. Our observation of a significantly superior improvement in memory retrieval for the MemFlex condition, but no downstream effect to depressive relapse, may suggest that impaired memory retrieval is not such a strong predictor of prognosis in those who complete depressive treatments. Further exploration of this issue is necessary to improve understanding of cognitive risk factors for recurrence of depression.

Further development of low-intensity interventions for reducing depressive relapse will improve intervention options available for individuals, and ultimately help to alleviate the burden of recurrent depression. MemFlex may improve some cognitive risk factors for depression, above the effect of Psychoeducation. However, the negligible effect sizes we observed on depressive outcomes suggest that MemFlex may be most effectively used as a treatment program, rather than as a relapse prevention program.

## Trial registration

ClinicalTrials.gov, Identifier NCT02614326.

## Published protocol paper

Hitchcock, C., Gormley, S., O'Leary, C., Rodrigues, E., Wright, I., Griffiths, K., … Dalgleish, T. (2018). Study protocol for a randomized, controlled platform trial estimating the effect of autobiographical Memory Flexibility training (MemFlex) on relapse of recurrent major depressive disorder. *BMJ Open, 8*(1). https://doi.org/10.1136/bmjopen-2017-018194.

## CRediT authorship contribution statement

**Caitlin Hitchcock:** Conceptualization, Data curation, Formal analysis, Funding acquisition, Investigation, Methodology, Project administration, Resources, Supervision, Visualization, Writing – original draft, Writing – review & editing. **Alicia J. Smith:** Data curation, Investigation, Methodology, Project administration, Writing – original draft, Writing – review & editing. **Rachel Elliott:** Data curation, Investigation, Methodology, Project administration, Writing – review & editing. **Cliodhna O'Leary:** Investigation, Data curation, Project administration, Writing – review & editing. **Siobhan Gormley:** Investigation, Data curation, Project administration, Writing – review & editing. **Jenna Parker:** Investigation, Data curation, Project administration, Writing – review & editing. **Shivam D. Patel:** Investigation, Data curation, Project administration, Writing – review & editing. **Carlos V. Esteves:** Investigation, Data curation, Project administration, Writing – review & editing. **Evangeline Rodrigues:** Investigation, Data curation, Project administration, Writing – review & editing. **Emily Hammond:** Resources, Writing – review & editing. **Peter Watson:** Formal analysis, Writing – review & editing. **Aliza Werner-Seidler:** Resources, Writing – review & editing. **Tim Dalgleish:** Conceptualization, Funding acquisition, Methodology, Resources, Supervision, Visualization, Writing – original draft, Writing – review & editing.

## Declaration of competing interest

The authors wish to declare that they were involved in the development of the MemFlex intervention evaluated in this trial, and in the tailoring of the Psychoeducation intervention from existing protocols.
