# Supplementary material for: A randomized, controlled proof-of-concept trial evaluating durable effects of memory flexibility training (MemFlex) on autobiographical memory distortions and on relapse of recurrent major depressive disorder over 12 months
Source: Behav Res Ther. 2021 May;140:103835. doi: 10.1016/j.brat.2021.103835 (PMC8047774; doi:10.1016/j.brat.2021.103835)
Supplement: Multimedia component 1 [file mmc1.docx]

**SUPPLEMENTARY MATERIALS**

**A randomised, controlled platform trial evaluating durable effects of Memory Flexibility Training (MemFlex) on autobiographical memory distortions and relapse of recurrent Major Depressive Disorder over 12 months**

Caitlin Hitchcock ^1*^

Alicia J. Smith^1^

Rachel Elliott^1^

Cliodhna O’Leary^1^

Siobhan Gormley ^1^

Jenna Parker ^1^

Shivam D. Patel^1^

Carlos V. Esteves^1^

Evangeline Rodrigues^1^

Emily Hammond ^1,3^

Peter Watson ^1^

Aliza Werner-Seidler ^2^

Tim Dalgleish ^1, 4^

^1^ Medical Research Council Cognition and Brain Sciences Unit, University of Cambridge

^2^ The Black Dog Institute, UNSW, Sydney

^3^ University of Exeter

^4^  Cambridgeshire and Peterborough NHS Foundation Trust

*Corresponding author: Tel +44 (0)1223 273 744, [Caitlin.hitchcock@mrc-cbu.cam.ac.uk](mailto:Caitlin.hitchcock@mrc-cbu.cam.ac.uk)

*Supplementary Table 1.* Intent-to-treat mean (standard error) proportion correct for the cognitive target and mean (standard error) performance on process measures, for each assessment, by intervention condition.

|  | MemFlex | | | | Psychoeducation | | | |
| --- | --- | --- | --- | --- | --- | --- | --- | --- |
|  | Baseline | Post | 6m | 12m | Pre | Post | 6m | 12m |
| AMT-AI Total | .67 (.03) | .84 (.02) | .77 (.03) | .87 (.03) | .69 (.03) | .80 (.02) | .74 (.03) | .76 (.03) |
| Specific | .76 (.05) | .91 (.03) | .87 (.04) | .88 (.04) | .74 (.05) | .87 (.03) | .80 (.04) | .78 (.05) |
| Categoric | .56 (.04) | .74 (.05) | .72 (.06) | .81 (.05) | .62 (.04) | .72 (.05) | .66 (.05) | .79 (.05) |
| Alternating | .68 (.03) | .84 (.03) | .77 (.03) | .85 (.05) | .69 (.03) | .79 (.03) | .76 (.03) | .74 (.05) |
| MEPS means | 4.24 (0.33) | 4.84 (0.41) | 4.82 (0.42) | - | 3.73 (0.31) | 4.81 (0.39) | 5.65 (0.41) | - |
| MEPS efficacy | 6.45 (0.46) | 6.29 (0.41) | 6.18 (0.48) | - | 6.05 (0.44) | 6.31 (0.39) | 6.43 (0.46) | - |
| Verbal fluency errors | 1.68 (0.34) | 1.82 (0.54) | 1.32 (0.31) | - | 1.82 (0.54) | 0.94 (0.50) | 1.51 (0.30) | - |
| Rumination | 50.12 (1.99) | 47.76 (2.21) | 42.34 (1.85) | 45.36 (3.21) | 52.14 (2.01) | 48.85 (2.09) | 46.23 (1.78) | 48.71 (3.29) |
| Cognitive avoidance | 61.63 (3.35) | 58.50 (2.92) | 55.84 (3.20) | 51.63 (3.26) | 55.97 (3.34) | 52.57 (2.85) | 52.67 (3.10) | 52.50 (3.26) |

*Note.* AMT-AI= Alternating Instructions Autobiographical Memory Test; Categoric = Categoric block of AMT-AI; Specific= Specific block of AMT-AI; Alternating = Alternating block of AMT-AI; MEPS= Means Ends Problem Solving task; Verbal fluency errors= number of errors on the Verbal Fluency Task; Rumination= score on the Ruminative Response Style Scale; Cognitive avoidance= score on the Cognitive Avoidance Questionnaire.
